# Two hidden taxa in the Japanese encephalitis vector mosquito, *Culex tritaeniorhynchus*, and the potential for long-distance migration from overseas to Japan

**DOI:** 10.1371/journal.pntd.0010543

**Published:** 2022-06-30

**Authors:** Satoru Arai, Ryusei Kuwata, Yukiko Higa, Yoshihide Maekawa, Yoshio Tsuda, Sudipta Roychoudhury, Arlene Garcia Bertuso, Tran Vu Phong, Nguyen Thi Yen, Tomoki Etoh, Akira Otuka, Masaya Matsumura, Takeshi Nabeshima, Keiko Tanaka Taya, Nobuhiko Okabe, Mutsuo Kobayashi, Kyoko Sawabe

**Affiliations:** 1 Center for Surveillance, Immunization, and Epidemiologic Research, National Institute of Infectious Diseases, Tokyo, Japan; 2 Faculty of Veterinary Medicine, Okayama University of Science, Ehime, Japan; 3 Department of Medical Entomology, National Institute of Infectious Diseases, Tokyo, Japan; 4 Department of Parasitology, College of Public Health, University of the Philippines Manila, Manila, Philippines; 5 Department of Medical Entomology and Zoology, National Institute of Hygiene and Epidemiology, Hanoi, Vietnam; 6 Saga Fruit Tree Experiment Station, Saga, Japan; 7 Koshi Research Station, Institute for Plant Protection, National Agriculture and Food Research Organization, Kumamoto, Japan; 8 Institute for Plant Protection, National Agriculture and Food Research Organization, Ibaraki, Japan; 9 Department of Virology, Institute of Tropical Medicine, Nagasaki University, Nagasaki, Japan; 10 Kawasaki City Institute for Public Health, Kanagawa, Japan; NIAID Integrated Research Facility, UNITED STATES

## Abstract

The *Culex vishnui* subgroups, particularly *Culex tritaeniorhynchus*, are considered the primary vectors of the Japanese encephalitis virus (JEV) in Asia. Recent molecular phylogenetic analyses of JEV isolates from Asian countries have shown that JEVs with diverse genetic variants are present in Asia. Furthermore, some JEV strains have been found to have crossed the East China Sea and been introduced into Japan. In this study, the possibility of overseas migration of the JE vector mosquito, *Cx*. *tritaeniorhynchus* was examined from the genetic, physical, and meteorological perspectives. Molecular phylogenetic analysis was performed based on both whole coding sequences and on the barcoding region of the mitochondrial cytochrome c oxidase subunit I (*COI*) gene of *Cx*. *vishnui* subgroups collected from Asian countries. *Culex tritaeniorhymchus* was classified into two genetically independent taxa by *COI* sequences: the Japanese type (*Ct*-J), which inhabits Japan except for the Amami Islands of southern Japan, and the continental type (*Ct*-C), which inhabits the Asian region except for Japan. It was confirmed that approximately 10% of *Cx*. *tritaeniorhynchus* trapped during the summer in western Kyushu were *Ct*-C, and that they could fly for up to 38 h continuously. The meteorological analysis also confirmed that the atmospheric flow occurring over the continent coincided with the date of *Ct*-C capture. This is the first report showing the existence of two taxa in *Cx*. *tritaeniorhynchus*. Their physical and physiological characteristics suggest the possibility of long-distance migration from overseas regions to Japan across the East China Sea. Future efforts are expected to provide evidence to support the occurrence of long-distance migration of *Cx*. *tritaeniorhynchus* with JEV.

## Author summary

We have been conducting research and studies on the importance of understanding the biological characteristics of each vector species in order to eradicate vector-borne diseases. In recent years, most reported cases of mosquito-borne diseases in Japan, such as dengue fever, malaria, and chikungunya fever, are imported cases from overseas; however, Japanese encephalitis (JE) is the only mosquito-borne disease for which autochthonous cases have been confirmed. The primary vector of JE virus (JEV) in Asia, a species called *Culex tritaeniorhynchus*, has long been recognized as a single species, however, this study is the first to report the existence of a cryptic species that is genetically distinct from *Culex tritaeniorhynchus*. Although long-distance migration of this species has been suspected for a long time, it has never been experimentally verified, and its contribution to the spread of JEV has not been investigated. The present study is also the first to confirm the capability of long-distance migration of this species biologically and physically, and to support it with meteorological data. Our objectives are to contribute to the prediction and minimization of the JE epidemic in Asia based on the results of experiments using the latest molecular genetic techniques combined with detailed physiological investigations.

## Introduction

Japanese encephalitis (JE), the main cause of viral encephalitis, is an important human infectious mosquito-borne disease, with an estimated 68,000 clinical cases reported globally each year, and more than 30% of the cases result in death every year [1]. The occurrence of JE virus (JEV; family, Flaviviridae; genus, Flavivirus) has been reported throughout Asia [2–4], and this virus has recently spread to South India, Sri Lanka, and Northern Australia [5–7]. Twenty-four countries in the World Health Organization (WHO) South-East Asia and Western Pacific regions have endemic JEV transmission, and it has been estimated that more than three billion people are at risk of JEV infection [1]. Outside of these endemic areas, the emergence of JEV in Angola was reported in 2016 [8]. In Italy, viral RNA was detected in birds in 1997–2000 and *Culex pipiens* mosquitoes in 2010 [9,10]. JEVs have been isolated from more than 30 different species of mosquitoes, mainly the genus *Culex* [4]. However, three of these species, *Cx*. *vishnui*, *Cx*. *pseudovishnui*, and *Cx*. *tritaeniorhynchus*, which belong to the *Cx*. *vishnui* subgroup, have been recognized as the most important vectors of JEV in endemic countries. One of the main characteristics of this group is that the morphological features among its members are so similar that it is difficult to distinguish between species based on morphological classification alone. It is also recognized that among the members of this subgroup, it has been recognized that *Cx*. *tritaeniorhynchus* is arguably the most important JEV vector throughout Asia.

In Japan, a large JEV epidemic occurred in 1924, with more than 6,000 cases, and more than 60% of these cases resulted in death [11]. Vaccination against JEV began in the latter half of the 1960s. The number of patients infected with JEV has declined rapidly, with fewer than 10 cases per year since 1999 [12]. Furthermore, the management of paddy fields, which represent the main habitat of *Cx*. *tritaeniorhynchus* larvae, and breeding practices for domestic animals, mainly pigs, have undergone extensive changes. Despite the decrease in JE cases due to these factors, the JEV becomes active every summer in both amplifying animals and vector mosquitoes [13,14]. Therefore, as the virus is still active, opportunities for autochthonous infections are not lost. Currently, whether JEV overwinters in Japan during the winter or migrate from overseas during the summer is not well understood. Based on the analysis of JEV strains isolated in Asian countries, it was recently observed that virus strains with diverse genetic variations are present throughout Asia. For example, JEV strains isolated from Vietnam and China were found in Japan a few years later [15–17]. These findings strongly suggest that some JEV strains have been introduced into Japan from Southeast Asia. How these JEVs are brought into Japan remains an important question.

In the late 1960s, variety of insects including agricultural insects were captured on the weather-ship Tango in the East China Sea (29 N and 135 E), approximately 500 km off the islands of Japan [18–20]. Based on these findings, rice planthoppers, such as brown planthopper, *Nilaparvata lugens*, white-backed planthopper, *Sogatella furcifera*, and small brown planthopper, *Laodelphax striatellus*, were identified to frequently migrate to Japan from overseas each year [21]. As planthoppers are known to be harmful insects that damage and transmit viral disease to rice plants, studies to predict their migration patterns from continental Asia began to rapidly develop in the 1980s. Currently, models using highly precise three-dimensional simulations are being developed [22–24]. In this system, the migration dates of planthoppers were estimated based on the presence of a strong wind level of 850 hPa on the weather charts. This prediction program was developed by the Japan Plant Protection Association (JPP-NET) and is already in general use. During the survey in the late 1960s, several *Cx*. *tritaeniorhynchus* mosquitoes were identified among the insects captured at sea [18–20]. Furthermore, in Jiangsu Province, China, *Cx*. *tritaeniorhynchus* has also been observed returning from the north in October, covering an estimated distance of 200 km per night [25]. These studies provide evidence of long-distance migration of *Cx tritaeniorhynchus*. As such, the flight range of vector mosquitoes is an important factor in predicting the risk area of transmission of mosquito-borne pathogens to humans. It is necessary to physically verify the flight capability of this species. However, although there have been some reports of studies on forced flight experiments for *Aedes* mosquitoes [26–29] and *Culex pipiens pallens* [30], no studies have been conducted on *Cx*. *tritaeniorhynchus*.

In *Cx*. *tritaeniorhynchus*, a notable variant of the var. *siamensis* has been previously described based on morphological characteristics [31,32]. This variant was later recognized as a subspecies, *Cx*. *tritaeniorhynchus summorosus* [33,34] or variant [35]. Recently, *Cx*. *tritaeniorhynchus summorosus* was considered a variety of the subspecies *Cx*. *tritaeniorhynchus*, specifically in the Indian strain [36]. Today, genetic analysis such as using the mitochondrial cytochrome c oxidase subunit I (*COI*) have become popular, but results applied to this species have not yet been reported. Therefore, according to conventional taxonomy, the common perception has been that *Cx*. *tritaeniorhynchus* is a single species, though it is widely distributed throughout Asia. In this study, phylogenetic analysis was conducted based on COI sequences of *Cx*. *tritaeniorhynchus* collected in Asian countries, including Japan, and the results indicated that they can be divided into two genetically different taxonomic groups. We used the above indices to differentiate between mosquitoes captured using net traps for planthoppers in the Kyushu region during the summer season, and it was confirmed that some mosquitoes had flown from abroad. We conducted a forced flight experiment using a flight mill [37,38] and a meteorological analysis using the National Oceanic and Atmospheric Administration’s (NOAA) Hybrid Single Particle Lagrangian Integrated Trajectory Model (NOAA based on meteorological analysis using the NOAA HYSPRIT model [39], to estimate the flight capabilities of the *Cx*. *tritaeniorhynchus* and the possibility of their participation in long-distance migration.

## Methods

### Mosquito collection

Mosquitoes were collected in Japan, Vietnam, Philippines, and Indonesia from 2007 to 2011. Adult mosquitoes were collected around livestock throughout the day using a Centers for Disease Control and Prevention (CDC) miniature light trap enhanced with dry ice throughout the day [40]. The aspirators were generally used to collect mosquitoes on livestock for 3 h each night between 18:00 and 21:00. Collected adult mosquitoes were transported in an icebox to the laboratory of each counterpart. Larval mosquitoes were collected from paddy fields using dippers. Larvae were transported alive to the office and reared to adults under laboratory conditions, then identified after their emergence. All field-collected adult mosquitoes were transported on ice to the laboratory for species identification according to established identification keys [41–44]. Following species identification, the mosquitoes were stored at −80°C until DNA extraction. All classified mosquito specimens were transferred individually into 1.8 mL microtubes (Eppendorf, Hamburg, Germany), and stored at -80°C until subsequent analyses by *COI* sequencing. The collectors always wore long-sleeved shirts, long pants, and hats, and applied repellent to the bare skin of their hands and faces to prevent mosquito bites.

The net trap (NT) and Johnson-Taylor suction trap (JT-ST) (Burkard Manufacturing Co. Ltd., Hertfordshire, UK) have often been employed as standard tools in agricultural investigations for monitoring long-distance migratory insects. Our NT consists of a net attached to a 1.5 meter deep, 1 meter diameter ring at the top of a pole 10 m above the ground. Insect collection using NTs was conducted at three locations in the western Kyushu area, namely, Saga, Iki, and Goto Cities. A JT-ST was used in Minami-Satsuma City, located in the southern Kyushu area. Mosquitoes trapped in NTs between June and September 2009 and 2010 were used for the analysis.

### Sequence analysis of genomic DNA of mosquito specimens

Total genomic DNA was extracted using the REDExtract-N-Amp Tissue PCR Kit (Sigma-Aldrich Co. LLC., St. Louis, MO, USA) or MagDEA DNA 200 (GC) (Precision System Science, Matsudo, Japan) according to the manufacturer’s protocol. Genomic DNA of mosquitoes was used to determine the nucleotide sequence of the entire 1,542-base-pair (bp) of the *COI* gene and the 658 bp of partial *COI* sequences, the DNA barcoding region [45,46]. The *COI* barcoding region was amplified using primer pairs LCO1490 and HCO2198 [45] and TaKaRa Ex Taq Hot Start Version (TaKaRa Bio Inc., Shiga, Japan) or a newly designed primer pair, MosMt-948F (5’-AGG WGG ATT ACC YCC ATT TYT AGG A-3’) and MosMt-3070R (5’-ATC CTA AAT TTG CTC AGG TTG CCA-3’) with the Phusion enzyme (New England Biolabs, Ipswich, MA, USA). The PCR reactions were carried out in a 10 μl volume containing 1.00 μl of 10x PCR buffer, 0.80 μl of 2.5 μM dNTP mixture, 0.05 μl of 5 U/μl Ex Taq HS or Phusion enzyme, 0.50 μl of each 2.5 μM primer, 6.15 μl of DDW and 1.00 μl of DNA template. Amplification conditions were as follows: initial denaturation at 95°C for 5 min, followed by 5 cycles of 94°C for 40 s (denaturation), 45°C for 1 min (annealing), 72°C for 1 min (extension), 35 cycles of 94°C for 40 s (denaturation), 51°C for 1 min (annealing), 72°C for 1 min (extension), and a final extension at 72°C for 10 min for Takara Ex Taq or stepdown program was used for the Phusion enzyme: initial denaturation at 95°C for 2 min was followed by two cycles each of denaturation at 95°C for 15 s, 2°C step-down annealing from 60°C to 50°C for 30 sec, and elongation at 68°C for 1 min 30 s, then 30 cycles of denaturation at 95°C for 15 sec, annealing at 55°C for 30 s, and elongation at 68°C for 1 min 30 s. PCR products were confirmed with MultiNA (SHIMADZU Corporation, Kyoto, Japan) and a DNA 12000 reagent kit. The resultant amplification products were purified with ExoSAP (Affymetrix Inc., Santa Clara, CA, USA) or Centri-Sep columns (Princeton Separations, Adelphia, NJ, USA). Sequencing samples were prepared using the BigDye Terminator ver1.1 Cycle Sequencing Kit (Thermo Fisher Scientific K.K., Tokyo, Japan), and the base sequences were decoded with ABI PRISM 3100-Avant Genetic Analyzer (Thermo Fisher Scientific K.K.) and edited with ATGC-Win ver.14 (Genetyx Corp., Tokyo, Japan).

### Phylogenetic analysis

Molecular phylogenetic analysis was performed based on the nucleotide sequences of the whole coding sequences and the barcoding region of the mtDNA *COI* gene. Multiple alignments of the sequences from selected mosquito strains were carried out using the Clustal W program [47]. GenBank accession numbers for the sequences used in the phylogenetic analysis are listed in S1 Table. The aligned matrix data were analysed using MrBayes software [48] for Bayesian phylogenetic inference under the Markov chain Monte Carlo algorithm with the GTR+I+G model evolution, as selected by jModelTest [49].

### Flight mill experiments

The mosquitoes employed in this analysis were of the *Cx*. *triteniorhynchus* Chiba strain (*Ct*-J) collected from Chiba Prefecture (35.60 N, 140.24 E) in 2009 and the *Cx*. *pipiens pallens* NIID strain (*Cpp*) collected from Tokyo Metropolis (35.42 N, 139.43 E) in 2008. Both strains were established as laboratory colonies at 25°C and 70% relative humidity (RH), under a photoperiod of 16:8 (Light:Dark) h, and maintained after emergence under three different combinations of photoperiod and temperature: 8L:16D and 15°C; 11L:13D and 20°C; and 16L:8D and 25°C, with 80% RH. The flight mill experiments were carried out under the same conditions used for mosquito maintenance. Ten females, 7–10 days after emergence, were used for the flight mill experiments. The experiments were replicated twice, in June 2010 and November 2011.

The rotor consisted of a thin steel line of 14 cm in total length as a horizontal arm, an insect pin of 4 cm as a rotating axis, and a small sponge rubber block to which the arm and the axis were fixed in a cross shape. Both outer parts of the arm were bent at 2 cm from each tip by 90° downward. The length of the right or left arm was approximately 5 cm, and the weight of the rotor was in average 81.6 mg. Twenty rotors were placed in an incubator and operated at 15°C, 20°C, and 25°C with 80% RH. Immediately before the flight mill experiment, the tip of the arm was attached to the notum of mesothorax of a mosquito lightly anesthetized by placing it on ice, with a small amount of quick-drying adhesive (Wood glue, Konishi Co., Ltd., Osaka, Japan). Each rotor with an insect was set in the up and down directions attached to two parallel acrylic rods. Two pulse electric signals per 360° rotation were produced by a photo sensor attached to the rods. The number of pulses per 5 s was recorded automatically by specify software; fewer than four pulses per 5 s did not meet a standard for continuous flight, and as a result, were not included in a later analysis. Flight mills were continuously monitored for at least 48 h. Thereafter, the continuous flight time for each individual mosquito was calculated. A *t*-test was used for the statistical analysis. Statistical significance was set at *p* <0.05. GraphPad Prism software version 7 and Microsoft Excel 2016 were used for the statistical analyses.

In this study, we could only evaluate the flight capability of *Ct*-J. Originally, we should have used a flight mill to estimate the flight capability of *Ct-*C. Unfortunately, as far as we know, there are no maintained colonies of *Ct*-C in the world. Although whether *Ct-*C can fly as well as *Ct*-J needs to be clarified in future studies using *Ct*-C, it is known that in late autumn, long-distance domestic migration of *Ct*-J occurs suddenly, which is thought to be a prediapause seasonal migration from breeding sites to overwintering sites in Japan [50]. It is reasonable to assume that this ability to migrate long distances is a common characteristic of *Cx*. *tritaeniorhynchus*. Therefore, we considered that the flight capability of *Ct*-C could be evaluated based on the results of flight-mill experiments using *Ct*-J.

### Backward trajectory analysis using the NOAA’s HYSPRIT model

HYSPLIT is a computer model used to compute air parcel trajectories and the deposition or dispersion of atmospheric pollutants. This tool was developed by NOAA and Australia’s Bureau of Meteorology [51,52]. HYSPLIT can be run in client-server mode from the NOAA website: https://www.ready.noaa.gov/HYSPLIT.php. The backward trajectory analysis of HYSPLIT can be combined with satellite imagery to infer how air pollution has been propelled by air masses. We analysed the atmospheric flow at 24–36 h before the day when *Cx*. *tritaeniorhynchus* was found, and determined a possible flying path from the continent to four locations in Kyushu District, western Japan, where NTs and JT-ST were installed.

## Results

### Phylogenetic analyses of members of the *Cx*. *vishnui* subgroup

A total of 86 mosquito specimens from the *Cx*. *vishnui* subgroup, comprised of 60 specimens from Japan and 26 from six Asian regions outside Japan, were used for phylogenetic analysis (Table 1).

**Table 1:** Mosquito specimens used in this study.

	Mosquito species	Origine	No. specimens analyzed		
			Total	*Ct*-C	*Ct*-J
***Culex vishnui* subgroup**					
	*Culex tritaeniorhynchus*	Japan	49	6	43
		South Korea	2	1	1
		China	2	2	0
		Taiwan	3	3	0
		Philippines	1	1	0
		Thailand	1	1	0
	* *	Vietnam	11	11	0
	*Culex pseudovishnui*	Japan	10	10	0
	*Culex vishnui*	India	1	1	0
		Japan	1	1	0
		Philippines	5	5	0
	Total		86	42	44
**Out group species**					
	*Aedes aegypti*		1		
	*Aedes albopictus*	Japan	1		
	*Anopheles darlingi*	Brazil	1		
	*Anopheles gambiane*		1		
	*Cnephia dacotensis*	Canada	1		
	*Culex bitaeniorhynchus*	Japan	1		
	*Culex decens*	Senegal	1		
	*Culex gelidus*	China	1		
	*Culex infantulus*	Japan	2		
	*Culex mimeticus*	Japan	1		
	*Culex nigropunctatus*	China	1		
	*Culex orientalis*	Japan	1		
	*Culex pipiens*	Japan	2		
	*Culex quinquefasciatus*		1		
	*Culex sasai*	Japan	1		
	*Culex tarsalis*	USA	1		
	*Culex torrentium*	Russia	1		
	*Culex vagans*	South Korea	2		
	*Culiseta impatiens*	Canada	1		
	*Tipula cockerelliana*	China	1		
	Total		23		

The details of the specimens were as followed. Sixty-nine *Cx*. *tritaeniorhynchus* including 19 sequences from GenBank database, 10 *Cx*. *pseudovishnui* including 5 from GenBank database and 7 *Cx*. *vishnui* including 2 from GenBank database. The MrBayes tree was constructed based on 1542 complete nucleotide sequences of the *COI* gene with 26 corresponding strains from the GenBank database. Twenty-three specimens were outgroup sequences (21 corresponding sequences from GenBank databases and two specimens analysed in this study) (S1 Table).

**Fig 1:**
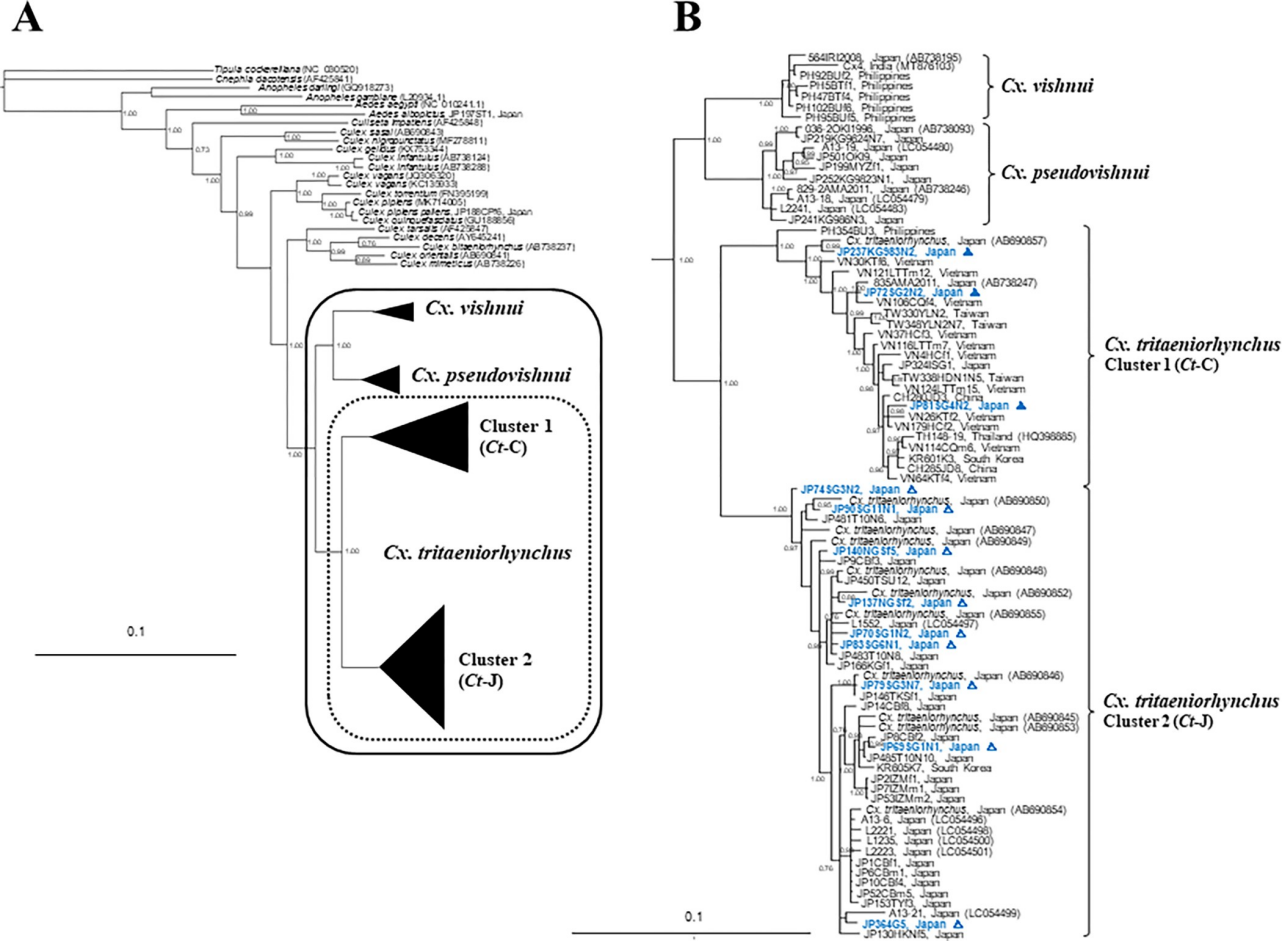
Bayesian inference of a phylogenetic tree based on the nucleotide sequences of the mitochondrial *COI* region (1542 bp) of 69 *Cx*. *tritaeniorhynchus* specimens. A: Summarized Bayesian phylogenetic tree with 86 *COI* sequences of *Cx*. *vishnui* subgroup and 23 sequences as an outgroup. The black frame indicates *Cx*. *vishnui* subgroup, and the dotted frame indicates *Cx*. *tritaeniorhynchus*. B: Expanded subtree of 86 *COI* sequences of *Cx*. *vishnui* subgroup. Bootstrap values correspond to 1000 replications. Bar denotes the nucleotide similarity distance. GenBank accession numbers for sequences used in the phylogenetic analysis are in S1 Table. Open and closed triangles with blue indicate *Ct*-J and *Ct*-C subtypes of the *Cx*. *tritaeniorhynchus* specimens, respectively, captured in net-traps (NTs) and a Johnson-Taylor suction trap (JT-ST).

**Fig 2:**
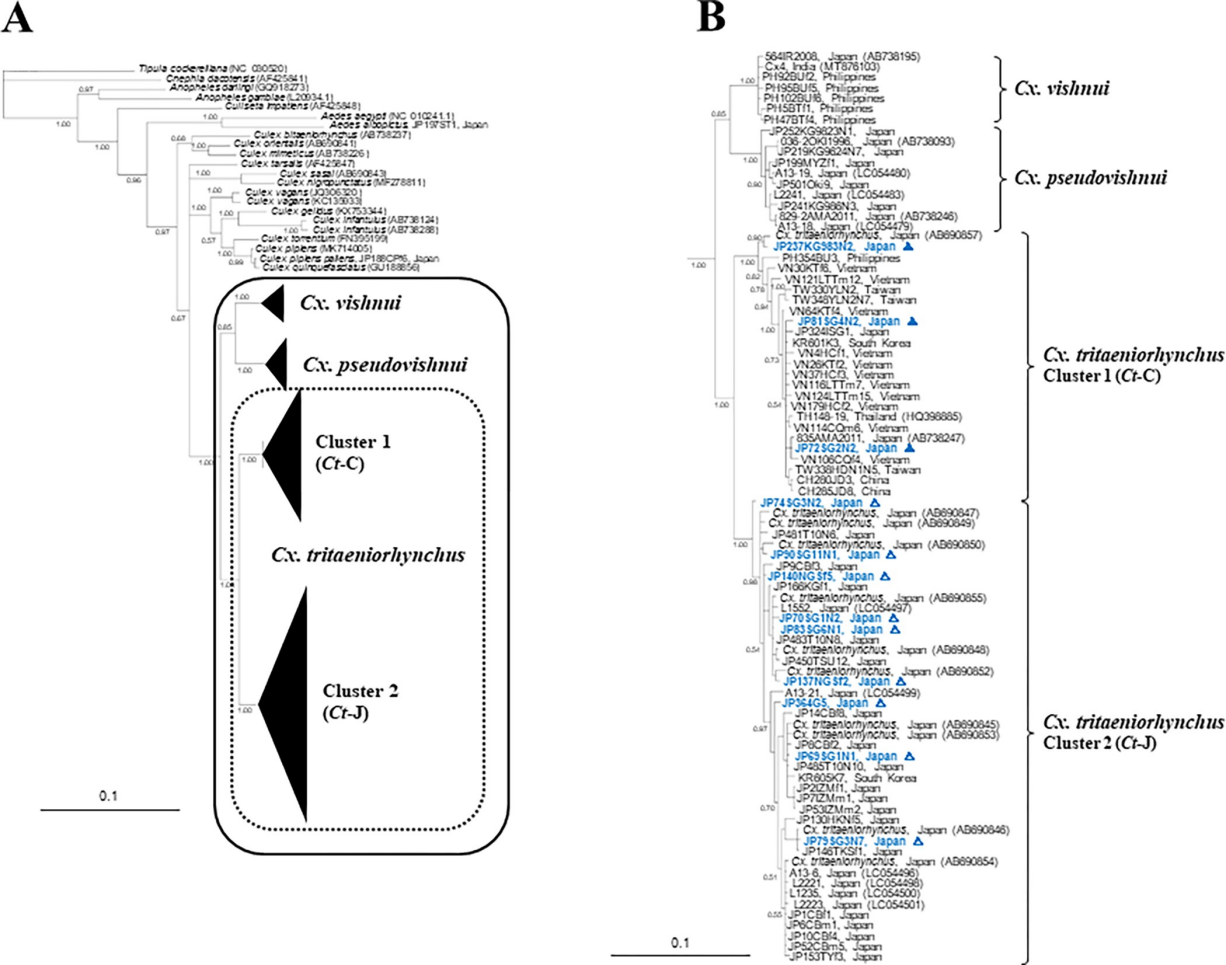
Bayesian inference of a phylogenetic tree based on the partial nucleotide sequences of the mitochondrial *COI* region (658 bp) of 69 *Cx*. *tritaeniorhynchus* specimens. A: Summarized Bayesian phylogenetic tree with 86 *COI* sequences of *Cx*. *vishnui* subgroup and 23 sequences as an outgroup. The black frame indicates *Cx*. *vishnui* subgroup, and the dotted frame indicates *Cx*. *tritaeniorhynchus*. B: Expanded subtree of 86 *COI* sequences of *Cx*. *vishnui* subgroup. Numbers at nodes indicate posterior probability values (>0.7) based on 150,000 trees: two replicate Markov chain Monte Carlo runs, consisting of six chains of 10 million generations each sampled every 100 generations with a burn-in of 25,000 (25%). Bar denotes the nucleotide similarity distance. GenBank accession numbers for sequences used in the phylogenetic analysis are in S1 Table. Open and closed triangles with blue indicate *Ct*-J and *Ct*-C of the *Cx*. *tritaeniorhynchus*, respectively, captured in net-traps (NTs) and a Johnson-Taylor suction trap (JT-ST).

The *Cx*. *vishnui* subgroup was classified into four robust taxa: *Cx*. *vishnui*, *Cx*. *pseudovishnui*, and two *Cx*. *tritaeniorhynchus* (Fig 1). The *Cx*. *tritaeniorhynchus* specimens were divided into two taxa. The phylogenetic relationship between these two taxa (clusters 1 and 2) was suggested to be as independent as that between *Cx*. *vishnui* and *Cx*. *pseudovishnui*. Cluster 1 of *Cx*. *tritaeniorhynchus* was composed of 19 specimens from overseas, but also included three specimens from Japan (two from Okinawa Prefecture and one from Kagoshima Prefecture). These three specimens were captured by NTs (two from Saga Prefecture) and a JT-ST (one from Kagoshima Prefecture). Cluster 2 of *Cx*. *tritaeniorhynchus*, consisting of 44 specimens, was composed entirely of 43 specimens from Japan, except for one specimen from South Korea. The remaining nine specimens captured by NTs were included in this cluster. The other MrBayes tree (Fig 2) was constructed based on 658 bp *COI* partial sequences of barcoding regions from the same specimens of the *Cx*. *vishnui* subgroup, as described previously. In this tree, a total of 22 specimens were outgroup sequences (20 corresponding sequences from GenBank databases, and two specimens analysed in this study) (S1 Table). There were four genetically independent taxa in the *Cx*. *vishnui* subgroup, which showed the same trend as the results shown in Fig 1. Surprisingly, even in a partial sequence of 658 nucleotides, this result showed that *Cx*. *tritaeniorhynchus*, previously thought to be a single species, encompasses two genetically distinct taxa.

**Fig 3:**
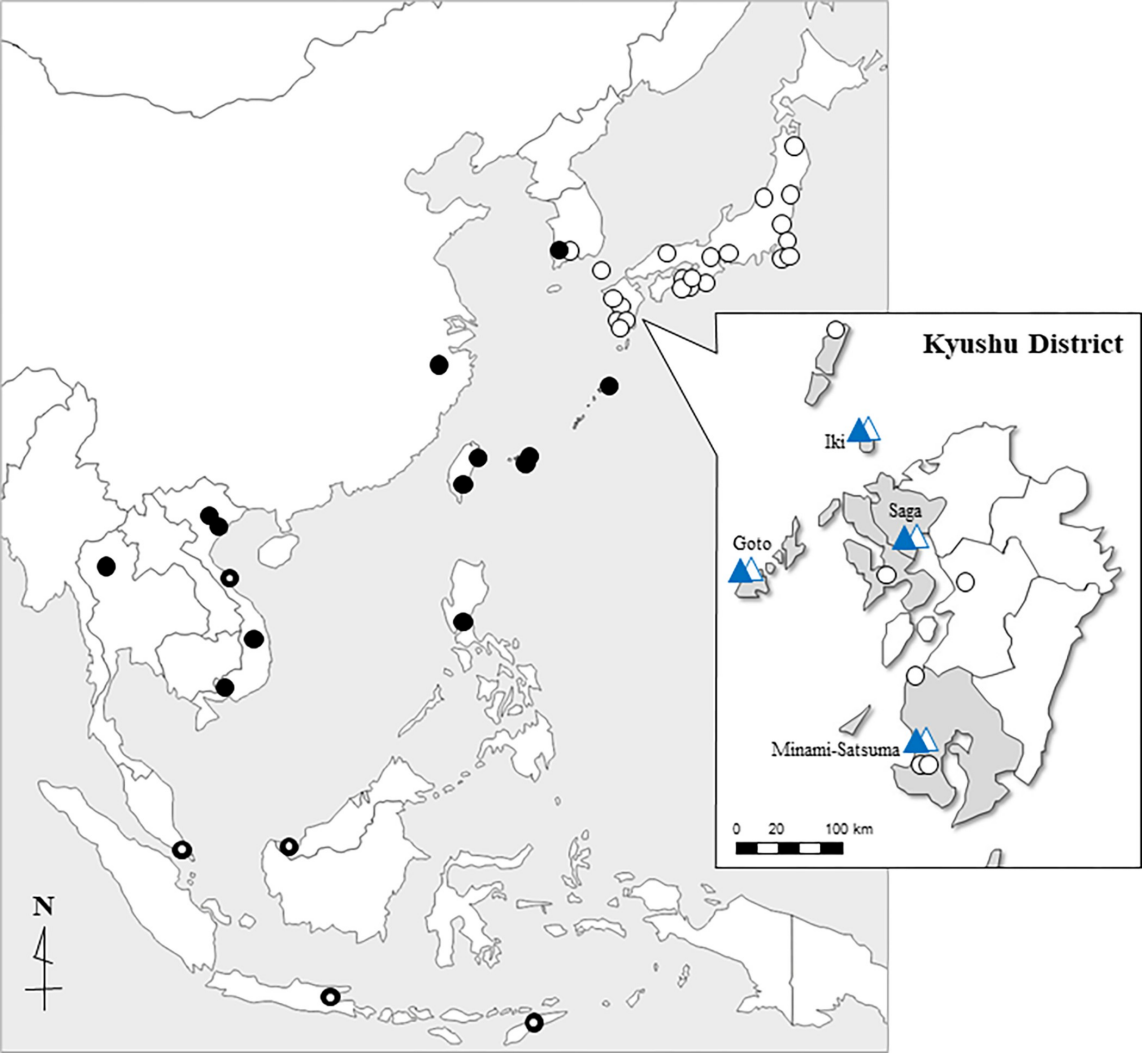
Locations of the mosquito collection sites in this study are shown here and details are given in S1 Table. The phylogenetic analysis indicated the *Cx*. *tritaeniorhynchus* was categorized into two taxa, *Ct*-J (open circles) and *Ct*-C (closed circles). The *Cx*. *tritaeniorhynchus* were captured in net-traps (NTs) and a Johnson-Taylor suction trap (JT-ST) installed at four localities on the Kyushu District (shaded area). Open and closed triangles with blue indicate capture of *Ct*-J and *Ct*-C of the *Cx*. *tritaeniorhynchus*, respectively. Double circles indicate specimens not used in the phylogenetic analysis, but are plotted on the map as the *Ct*-C referred from the *COI* sequences of the GenBank database. A map was created using free materials downloaded from the following websites: https://power-point-design.com/ppt-design/world-map-for-powerpoint/ and https://power-point-design.com/ppt-design/japan-map-available-for-powerpoint/. Based on the geographical positions recorded using a geographical positioning system (GPS: GPSMAP64, Garmin, USA) (S1 Table), the collection sites were plotted on this map.

Based on the results of these two phylogenetic trees, cluster 1, consisting of specimens from the south of the Amami Archipelago and Asian countries other than Japan, was designated as the continental type of *Cx*. *tritaeniorhynchus* (*Ct*-C), and cluster 2, consisting of specimens from northern mainland Kyushu, was designated as the Japanese type of *Cx*. *tritaeniorhynchus* (*Ct*-J; Fig 3). As shown in the callout of Fig 3, it is clarified that there are not only *Ct*-J but also *Ct*-C specimens in the summer. This means that there are times and seasons when these two types coexist in western Kyushu areas.

### Mosquitoes collected using net traps and Johnson-Taylor suction trap

Among the long-distance migratory insects captured between June 2009 and September 2010, a total of 1241 mosquitoes were trapped by NTs, and a JT-ST (Table 2). The mosquitoes were morphologically classified into three genera, namely, *Culex*, *Aedes*, and *Anopheles*, with five species. In 2009, a total of 32 and 222 specimens were captured in Saga and Minami-Satsuma Cities, while in 2010, 99 and 888 specimens were captured in Goto and Iki Cities, respectively. Among the captured specimens, *Cx*. *tritaeniorhynchus* was the most abundant species (47.8%). The 593 specimens of *Cx*. *tritaeniorhynchus* were classified into two types: *Ct*-C (11 specimens, 1.9%) and *Ct*-J (582 specimens, 98.1%) using the 658 bp *COI* partial sequences.

**Table 2:** Mosquitoes captured in net traps and a Johnson-Taylor suction trap in 2009 and 2010.

Mosquitoes	Years	2009		2010		Total	(%)
	Locations	Saga	Minami-Satsuma	Goto	Iki		
***Culex tritaeniorhynchus***		**27**	**81**	**14**	**471**	**593**	**(47.8)**
***Ct*-J**	** **	**24**	**80**	**12**	**466**	**582**	**(46.9)**
***Ct*-C**	** **	**3**	**1**	**2**	**5**	**11**	**(0.9)**
***Cx*. *pipiens pallens***		**4**	**3**	**4**	**175**	**186**	**(15.0)**
***Cx*. *quinquefasciatus***		**0**	**69**	**0**	**2**	**71**	**(5.7)**
***Cx*. *pseudovishnui***		**0**	**4**	**0**	**0**	**4**	**(0.3)**
***Culex* spp.**	** **	**0**	**19**	**2**	**0**	**21**	**(1.7)**
***Aedes vexans***	** **	**0**	**0**	**0**	**60**	**60**	**(4.8)**
***Aedes* spp.**	** **	**1**	**20**	**36**	**5**	**62**	**(5.0)**
***Anopheles* spp.**	** **	**0**	**20**	**0**	**106**	**126**	**(10.2)**
**Un-identified**	** **	**0**	**6**	**43**	**69**	**118**	**(9.5)**
** **	**Total**	**32**	**222**	**99**	**888**	**1,241**	**(100)**
**Planthoppers**	** **	**273**	**729**	**639**	**NC**	**1,641**	** **

Net traps (NTs) were used in the Western Kyushu, Saga, Goto and Iki Cities. Johnson-Taylor suction trap (JT-ST) was used in the south Kyushu, Minami-Satsuma City.

*Culex tritaeniorhynchus* was discriminated into Japanese type (*Ct*-J) and Continental type (*Ct*-C) by the partial *COI* sequences. The shaded area marks total number of planthoppers collected at each trap.

**Fig 4:**
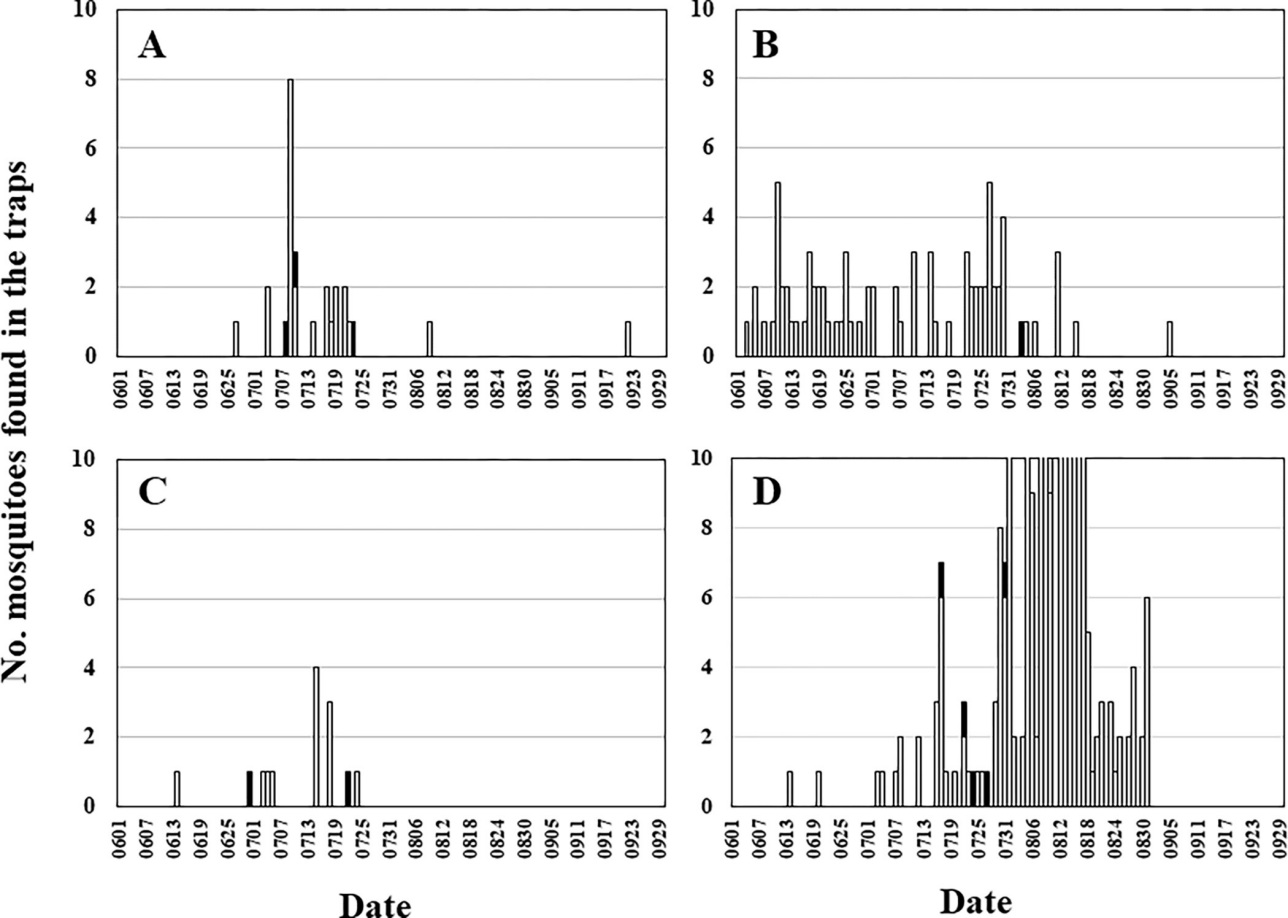
The days of *Cx*. *tritaeniorhynchus* capture in net-traps (NTs) and a Johnson-Taylor suction trap (JT-ST) installed at four localities on the Kyushu District. **A**: Saga City in 2009, **B**: Minami-Satsuma City in 2009, **C**: Goto City in 2010, **D**: Iki City in 2010. Open and solid bars indicate *Ct*-J and *Ct*-C subtypes of the *Cx*. *tritaeniorhynchus*, respectively, which were determined by the partial *COI* sequences (658 bp).

The discovery date of *Ct*-C between June and September is shown in Fig 4. In Saga City in 2009, three out of the 27 specimens were classified as *Ct*-C (11.1%). Each was found on July 8, 10 and 23. In Minami-satsuma City, only one *Ct*-C out of 81 specimens was captured, on 3 August 2009 (1.2%). In Goto City in 2010, one of the 14 *Ct*-C specimens was captured on 30 June and the other on 22 July (14.3%). In Iki City in 2010, one each of the 471 *Ct*-C specimens was captured on 17, 22, 24, 27, and 31 July (0.9%). The number of *Ct*-C specimens was particularly high in July in both 2009 and 2010. Of the total specimens captured, *Ct-*C specimens were 12.5% (3/24), 8.3% (1/12), and 11.1% (5/45) in the Saga, Goto, and Iki Cities, respectively. These results indicate that approximately 10% of the total trapped *Cx*. *tritaeniorhynchus* were *Ct*-C individuals that migrated to western Kyushu from overseas regions. When a JT-ST was used in Minami-Satsuma City in 2009, only one specimen of *Ct*-C was trapped on 1 August. This trend was different from that of the other three collection sites where NTs were installed.

### Flight capability of *Cx*. *tritaeniorhynchus* female based on flight mill experiments

**Fig 5:**
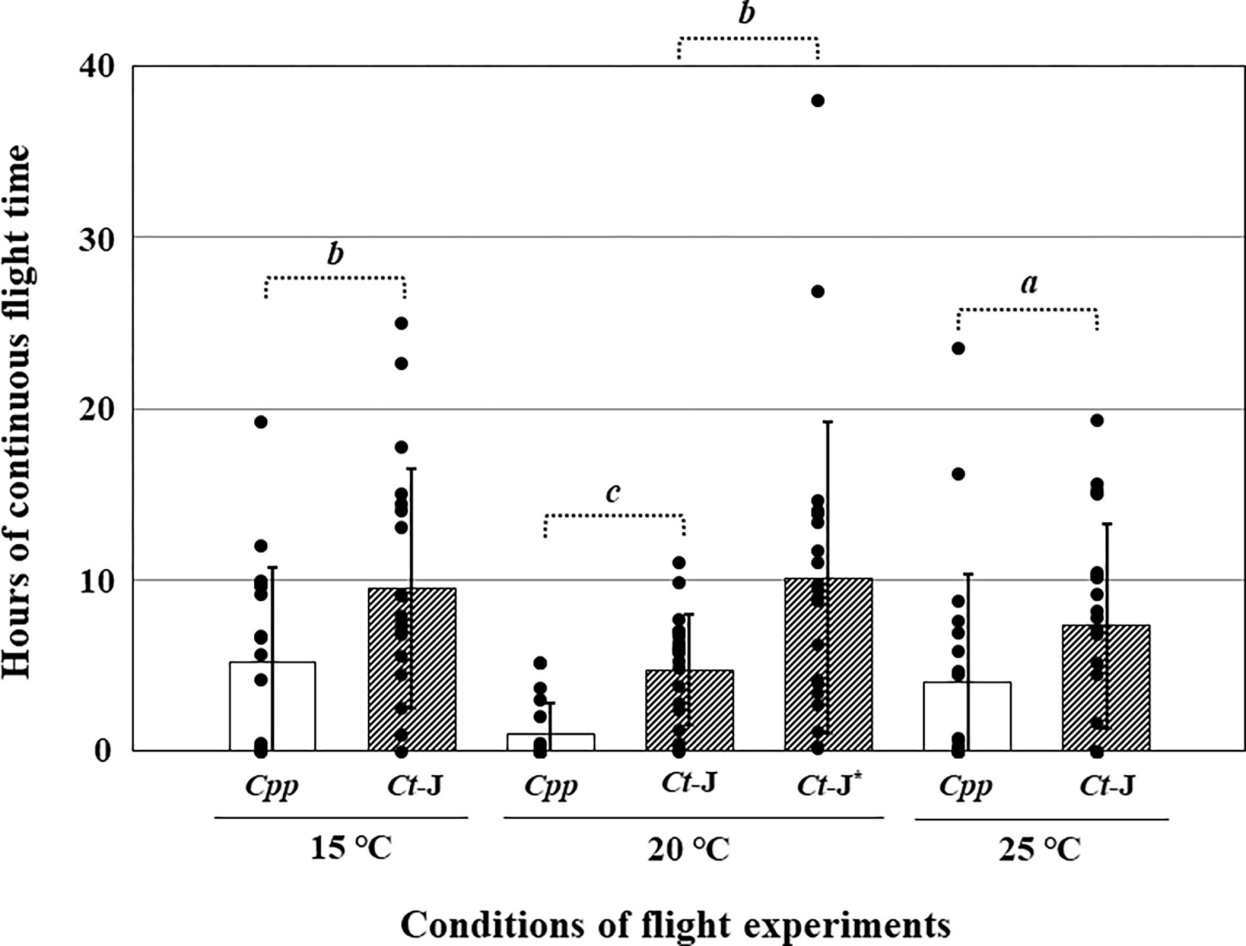
Comparison of a total flight time between *Cx*. *tritaeniorhynchus* (*Ct*-J, shaded bars) and *Cx*. *p*. *pallens* (*Cpp*, open bars) under each flight condition. The bars represent the mean with standard deviations (SDs). An independent two sample *t-*test assuming unequal variances was applied to determine significant differences. Statistically significant differences are indicated shown by Roman numerals; *a*, *p* < 0.001; *b*, *p* < 0.01; *c*, *p* < 0.1.

*Culex pipiens pallens* (*Cpp*) was the most abundant species caught in the NTs, after *Cx*. *tritaeniorhynchus*, in western and southern Kyushu (Table 2). Therefore, *Cpp* was considered the most suitable comparison in evaluating the flight capability of *Cx*. *tritaeniorhynchus*. Each 20 mosquitoes were used for the flight mill experiments. There was a large variation between individuals, and a few mosquitoes recorded no flight or less than three cycle rotations in 5 s. Therefore, the average flight times were calculated using mosquitoes that rotated over three cycles. These flight times were 10.05 h (0.95–25.05 h, n = 19) and 5.49 h (0.01–15.00 h, n = 19) at 15°C; 5 h (0.03–11.08 h, n = 19) and 1.28 h (0.01–5.2 h, n = 16) at 20°C; and 8.61 h (0.001–19.31 h, n = 17) and 4.45 h (0.02–16.18 h, n = 18) at 25°C for *Ct*-J and *Cpp*. The *Ct*-J specimens could fly longer than *Cpp* under all experimental conditions (*t*-test, *p* < 0.05 at 15°C, *p* < 0.001 at 20°C, and *p* < 0.1 at 25°C) (Fig 5). When the rearing and flight temperatures were the same, *Ct*-J had a longer continuous flight time than *Cpp* under all experimental conditions except *Ct*-J* at 20°C. *Ct*-J* females were reared at 25°C, but flight experiments were conducted at 20°C. Both species flew the longest at the lowest temperature of 15°C. Particularly *Ct*-J* at 20°C showed the longest total flight time at 10.14 h (0.2–37.97 h, n = 20). A significant difference was found between *Ct*-J and *Ct*-J* at 20°C (*t*-test, *p* < 0.05). Surprisingly, one *Ct*-J* specimen was found to fly continuously for 38 h.

**Fig 6:**
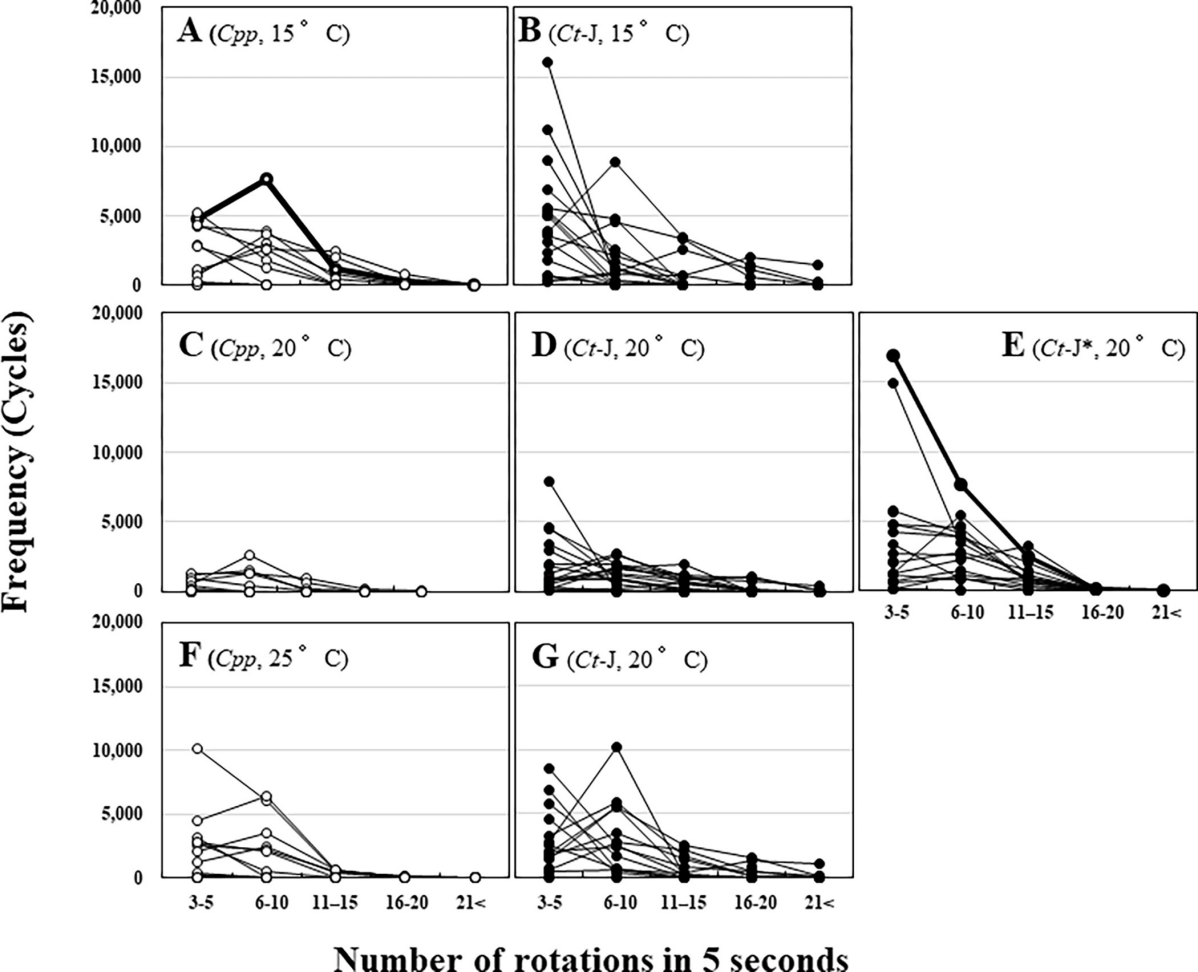
The frequencies of rotations within 5 s in the flight mill experiments between *Cx*. *tritaeniorhynchus* (*Ct*-J, closed circles) and *Cx*. *p*. *pallens* (*Cpp*, open circles). **A**: *Cpp* at 15°C, **B**: *Ct*-J at 15°C, **C**: *Cpp* at 20°C, **D**: *Ct*-C at 20°C, **E**: *Ct*-J* (*Ct*-J females were reared at 25°C, but flight experiments were conducted at 20°C) at 20°C, **F**: *Cpp* at 25°C, *Ct*-C at 25°C. Bold lines indicate the longest total flight time in each condition of *Cpp* at 15°C (**A**) and *Ct*-J* at 20°C (**E**).

**Fig 7:**
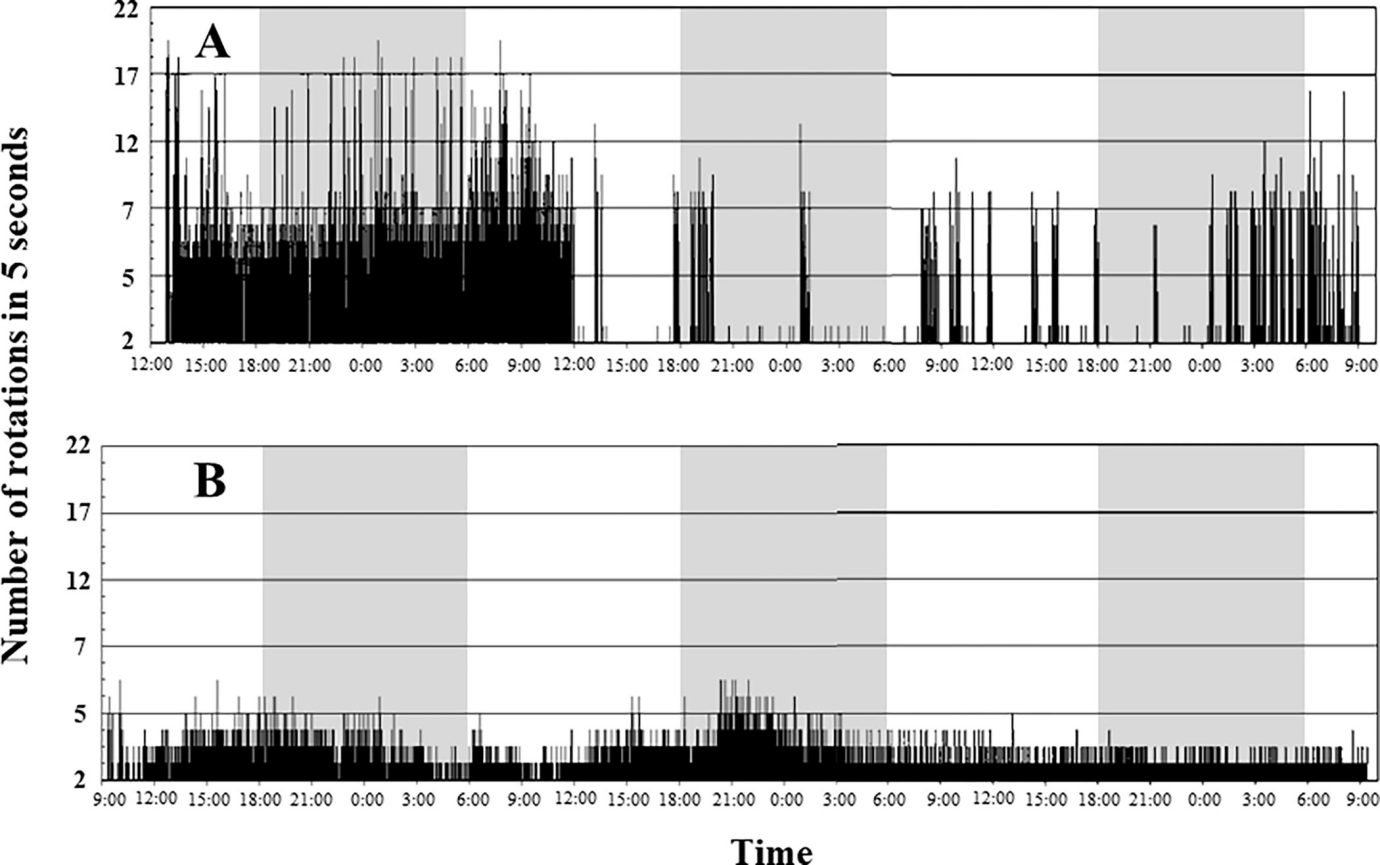
Comparison of the flight pattern between *Cx*. *p*. *pallens* (*Cpp*) at 15°C (A) and *Cx*. *tritaeniorhynchus* (*Ct*-J*) at 20°C (B). **A** and **B** are the flight patterns of the individuals with the longest total flight time in each experiment of Fig 6A and 6E, respectively. Flight patterns of *Cpp* and *Ct*-J* were observed for 69 h and 72 h, respectively. The shaded area marks environmental darkness.

Some of the *Cpp* specimens flew continuously for > 20 h; however, a remarkable difference in flight patterns was found between *Ct*-J and *Cpp*. The number of rotations in 5 s was compared (Fig 6). The results suggested that *Ct*-J tended to rotate for longer periods for a smaller number of rotations than *Cpp* under all conditions at 15°C (*Cpp* Fig 6A and *Ct*-J Fig 6B), 20°C (Fig 6C and 6D), and 25°C (Fig 6F and 6G). Particularly for *Ct*-J* (Fig 6E), three to five rotations were very common. Fig 7 shows the differences in flight patterns between each specimen that exhibited the longest flight time, *Cpp* (Fig 7A, shown in bold in Fig 6A) and *Ct*-J* (Fig 7B, shown in bold in Fig 6E). Although the *Cpp* fluttered their wings powerfully, it was for a short time, after which their wings tended to stop flapping immediately (Fig 7A). For *Ct*-J*, in contrast to *Cpp*, the flights lasted for a long period and their wings continued to flap very slowly throughout the flight (Fig 7B). The difference in flight patterns between *Cpp* and *Ct*-J* was more pronounced than the difference in the total flight distance.

### Backward trajectory analysis by the NOAA’s HYSPRIT Model

**Fig 8:**
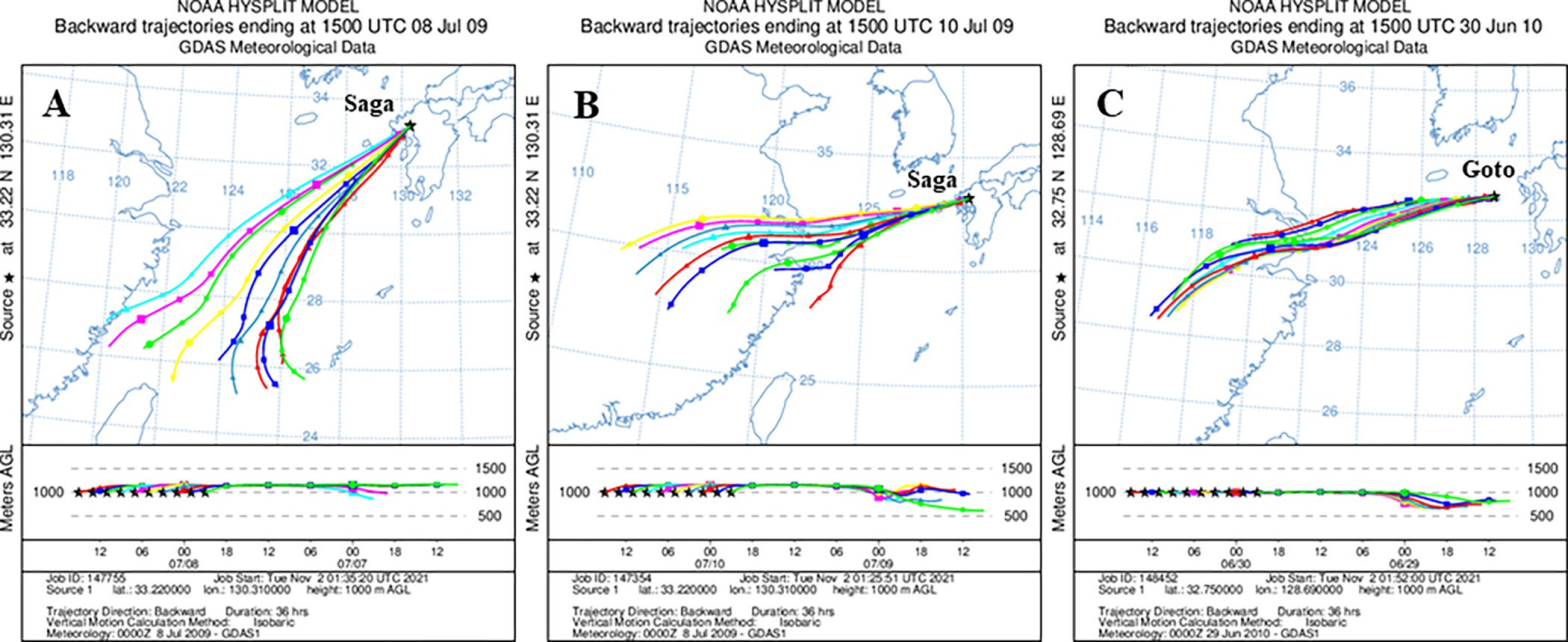
Computed back trajectories at 2-h intervals for the 36-h period ending at 1500 UTC of 8 and 10 July 2009 at Saga City (A and B), and 30 June 2010 at Goto City (C). Top portion shows the horizontal path, and the bottom portion shows the vertical path of the trajectories. Each figure is the result of a backward trajectory analysis of HYSPLIT using the NOAA website: https://www.ready.noaa.gov/HYSPLIT.php.

The results of the flight mill experiment showed that female *Ct*-J reared at 25°C flew the longest during the flight at 20°C (*Ct*-J*) (Figs 5 and 6E). This is important because the temperature at approximately 800–900 m above the ground is 20°C when the ground surface is approximately 25°C, and decreases by 0.6°C every 100 m higher. Therefore, we analysed the atmospheric flow in the airspace 1000 m above the ground. The distance between mainland China and western Kyushu is approximately 1000 km. It was suggested that the planthoppers could fly in the lower jet stream in the airspace around 1500 m above the ground [23,53]. Because the speed in this airspace is more than 10 m/s, it was calculated that the planthoppers were estimated to reach the western Kyushu areas within 24–36 h of departure. As the back trajectories in our study reached 1000 km within 36 h, we ran the HYSPLIT for 36 h alone. The HYSPLIT model was used to produce 10 back trajectories at 2-h intervals ending at 15:00 UTC on 8 July 2009 and 10 July 2009 in Saga City (Fig 8A and 8B), and on 30 June 2010 Goto City (Fig 8C). In these cases, the dates of *Ct*-C capture and the atmospheric flow coincided very well. The *Ct*-C specimens may have been carried by air flows originating from mainland China and the East China Sea up to 36 h before they arrived in Japan.

However, we found some cases where the date of *Ct*-C capture did not coincide with the onset of air flow. In these cases, air flows were observed from 36 h to several days earlier than the trap dates. The HYSPLIT model was used to produce 10 back trajectories at 2-h intervals ending at 00:00 UTC for each date. For example, it was estimated that the *Ct*-C specimen trapped in Saga City on 23 July 2009 may have been carried by air flows originating from South Korea or China on 21 July (S1A Fig), while the *Ct*-C specimen captured on 3 August 2009 in Minami-Satsuma City may have been carried by air flow from China on 29 July (S1B Fig), and the specimen captured on 13 July 2010 in Goto City may have been transported by air flow from China on 13 July (S1C Fig). In Iki City in 2010, it is possible that the *Ct*-C specimens captured on 17 July were carried by air flow from mainland China on 14 July (S1D Fig); those captured on 22 July likely departed from Korea on 15 July (S1E Fig); specimens captured on 24 and 27 July probably left Jeju Island, South Korea, on 26 and 27 July (S1F and S1G Fig), and those captured on 31 July most likely travelled on air flow generated in China on 30 July (S1H Fig). Considering the long-distance migratory planthoppers, as a reference, this data strongly suggested that the crossing of the East China Sea by *Cx*. *tritaeniorhynchus* is meteorologically possible.

## Discussion

In the present study, we demonstrated for the first time the existence of two taxa of *Cx*. *tritaeniorhynchus*, which are referred to as *Ct*-J and *Ct*-C in this study for convenience, using *COI* sequence analysis. In the *COI* phylogenetic trees developed in this study, the genetic distance between *Ct*-J and *Ct*-C was approximately the same as that between *Cx*. *vishnui* and *Cx*. *pseudovishnui*. From this result, we concluded that the *Cx*. *vishnui* subgroup consists of four species, instead of three. The present study also revealed that *Ct*-J and *Ct*-C are genetically distinct not only by the full-length *COI* sequence, but also by partial *COI* sequences. DNA barcoding using partial *COI* sequences can be used to identify closely related species, as well as new species. *COI* barcode region sequences of *Cx*. *tritaeniorhynchus* are now being accumulated, and the discussion of both types will proceed more quickly in the future. To date, the common perception has been that *Cx*. *tritaeniorhynchus* is a single species, though it is widely distributed throughout Asia, according to conventional taxonomy. However, our results strongly suggest the possibility of the existence of a cryptic species or a new species in *Cx*. *tritaeniorhynchus* based on the genetic aspects. Although we compared the morphology of the male genitalia, one of the most important taxonomic keys, between *Ct*-J and *Ct*-C, unfortunately, any significant differences were found. It is expected that the morphological characteristics of both types will be studied in detail in the future.

The present study revealed the existence of C*t*-J and C*t*-C in Korea, and suggested the Amami Archipelago could be the boundary of the distribution of C*t*-J and C*t*-C. However, the actual boundary of distribution between the two types is unknown. In addition, although we could not confirm any sequences on the *COI* gene showing the characteristics of hybridization between the two types, we cannot deny the possibility of the existence of hybrid, as they coexisted in the same region, even temporarily. As a next step, it will be necessary to analyse not only the mitochondrial genes from the mother, but also the nuclear genes inherited in pairs from both parents. Based on these facts and questions, we hope that as soon as possible a discussion will begin on whether to support each type as an independent species.

*Culex tritaeniorhynchus* is a tropical/subtropical mosquito that does not have a high resistance to low temperatures. However, this species has previously been observed to overwinter in caves in the Izu Peninsula and Chiba Prefecture in eastern Japan [54], and sewage infrastructure (such as culverts), even in urban areas of Japan [50]. Owing to global warming, the areas in which *the Cx*. *tritaeniorhynchus* can overwinter have undeniably expanded; however, the areas in Japan where they can overwinter remain limited. Therefore, there is a high possibility of *Cx*. *tritaeniorhynchus* in Japan is temporarily mixed with individuals that migrate from the south every year. Several findings have been revealed regarding the long-distance migration of *Cx*. *tritaeniorhynchus*. In Jiangsu province, China, they have been observed flying back from the north in October, covering an estimated distance of 200 km per night [25]. Recently, sequence divergence results have shown that the Australian population of this species is likely to have originated in East Timor (99.7% nucleotide similarity) [55]. These results suggested that while the introduction pathways were unconfirmed, it is plausible that *Cx*. *tritaeniorhynchus* may have travelled to Australia from Timor-Leste via windblown adult mosquitoes, given the relatively short distance (465 km) between Timor-Leste and Melville Island near Darwin, and that *Cx*. *tritaeniorhynchus* has been previously recorded as flying 200–500 km over sea waters in the Northwest Pacific [55]. These findings support the hypothesis that *the Cx*. *tritaeniorhynchus* has ample potential to migrate across the East China Sea into Japan.

We were able to detect *Ct*-C migrating from overseas to Japan using the *COI* sequence in this study. It was clarified that *Ct*-C was also captured in the deployed NT and JT-ST. A total of 11 *Ct*-C specimens were trapped from June 2009 to August 2010 in the Kyushu region. In these areas, approximately 10% of the specimens captured in the traps, especially in July, were *Ct*-C. Interestingly, the dates when *Ct-*C were caught coincided well with the predicted migration date of the planthoppers [56]. The following analysis was conducted under the assumption that this species has the same capabilities as planthoppers. A backward trajectory analysis using the HYSPRIT model in an airspace 1000 m above the ground was conducted, based on the weather data provided by the NOAA in the USA. The following three cases suggested that the migration date of the *Ct*-C and air flow coincided very well: specimens caught on 8 and 10 July in 2009 at Saga City, and on 30 June in 2010 at Goto City. In other cases, the migration of *Ct*-C did not coincide with air flow, but air flows that occurred before more than 36 h were observed. This suggests that C*t*-C, which had already arrived in Japan and stayed for a while, may have been captured in the trap along with C*t*-J.

The distance between mainland China and western Kyushu is approximately 1000 km. It was suggested that the planthoppers could fly in the lower jet stream in the airspace around 1500 m above the ground [23,53]. Because the speed in this airspace is more than 10 m/s, it is calculated that the planthoppers are estimated to reach the western Kyushu areas within 24–36 h. In our flight mill experiments, we simulated long-distance migration at 1000 m above the ground when the ground temperature was 25°C (*Ct*-J*). In other words, a female *Ct*-J reared at 25°C and flown at 20°C was observed to be able to fly continuously for approximately 38 h (*Ct*-J*). In addition, two females of *Ct*-J were also observed continuously flying at 15°C for up to 20 h (maximum 25 h). This result suggests that *Ct*-J can fly not only at 1000m above the ground, but also at 1500m in the airspace.

In this study, we only evaluated the flight capability of *Ct*-J. Although whether *Ct*-C can fly as well as *Ct*-J needs to be clarified in future studies using *Ct*-C, it is known that in late autumn, long-distance domestic migration of *Ct*-J occurs suddenly, which is thought to be a prediapause seasonal migration from breeding sites to overwintering sites in Japan [50]. It is reasonable to assume that this ability to migrate long distances is a common characteristic of *Cx*. *tritaeniorhynchus*. When the same mechanism of long-distance migration as that of the planthoppers can be applied to *Cx*. *tritaeniorhynchus*, then it is possible this species could physically migrate for more than 1000 km.

Previous reports on flight mill experiments in *Aedes* mosquitoes [26–29] and *Cx*. *p*. *pallens* (*Cpp*) [30] are known. For instance, the mean flight time for *Cpp* was 17,090.84 s (4.8 h) in 20-d-old females, and 14884.96 s (4.1 h) in 2-d-old. They also reported that their total flight time and distance tended to be shorter for 5- and 6-d-old females than any other age group, showing significant differences in flight capability between ages. This result suggested that young or aged females may be able to fly longer [30]. In our study using 7–10-d-old females, the longest flight time of *Cpp* was 5.49 h at 15°C. Other d-old *Cpp* may be able to fly for a longer time. Although there was no information on the *Cx*. *tritaeniorhynchus* in the previous study [30], it would also be necessary to evaluate the flight capability of young or aged females.

At the start of the 1990s, JEV in Asian countries, including Japan, transitioned from genotype III (GIII) to genotype I (GI). Currently, GI is still the major endemic strain at least in China and Japan [57,58]. However, in recent years, GV JEV has been isolated from *Cx*. *pipiens* group from China [59]. Furthermore, in South Korea, GV JEV has also been isolated not only from *Cx*. *Tritaeniorhynchus*, but also from several culicine mosquitoes, such as *Cx*. *pipiens* group, *Cx*. *bitaeniorhynchus*, and *Cx*. *orientalis*, which prefers avian over human [60]. A shift from GI to GV may have already begun in some areas of Asia. Every year, JEV becomes active in the summer in Japan; however, its ecology during winter is virtually unknown. Whether JEV overwinters in Japan or migrates each year from abroad has long been debated, and this issue has not yet been resolved. Nevertheless, based on phylogenetic analyses carried out in recent years, viruses from abroad have been found in Japan. It is very possible that viruses native to Japan overwinter in wild animals, such as wild boars or other mosquito species, in addition to *Cx*. *tritaeniorhynchus*. In contrast to this, our results support the hypothesis that *Cx*. *tritaeniorhynchus* migrates annually from the continent to Japan via a low-level jet stream. It is highly likely that such mosquito migration will result in simultaneous changes in JEV genotypes in Asia. Several issues remain to be clarified regarding the two taxa within *Cx*. *tritaeniorhynchus*, which provided the basis for this study, including the distributional boundaries of the two types, the existence of hybrid offspring between the two types, the ability of *Ct*-C to migrate long distances, the possibility of *Ct*-C overwintering in Japan, and the JEV prevalence of the two types. We hope that future efforts will provide evidence to support the long-distance migration of *Cx*. *tritaeniorhynchus* with JEV.

## Conclusions

This is the first report showing the existence of two genetically independent taxonomic groups in *Cx*. *tritaeniorhynchus*. The physical and physiological characteristics of *Cx*. *tritaeniorhynchus* suggests the possibility of long-distance flight from overseas regions, while meteorological studies confirmed the presence of atmospheric currents that make this possible. The epidemic strain of JEV might be influenced by several environmental conditions, including the weather phenomena of that particular year and the current epidemic strains of JE present in the regions of Asia, which might be the areas from which the mosquitoes migrate. It is thus important to consider multiple factors to effectively manage this disease, including the environment, vector mosquitoes, wild animals, humans, and the virus. In the future, we expect research to provide more information that will facilitate the management of this disease. For instance, summing up the results of surveys and studies from various fields, such as epidemiology and genetic information of JEV, mosquito ecology and physiology, and meteorology in Asia, will undoubtedly contribute to the prediction and control of JEV epidemics.

## Supporting information

S1 Table Details of the mosquito specimens used for phylogenetic analysis in this study. The geographical positions of the collection sites were obtained both from database and the current study. The geographic positions obtained in this study were recorded using a geographical positioning system (GPS: GPSMAP64, Garmin, USA).
(XLSX)

S1 Fig Computed back trajectories at 2-h intervals for the 36-h period. Computing was at 0000 UTC of 21 July 2009 at Saga City (**A**), 29 July 2009 at Minami-Satsuma City (**B**), 13 July 2010 at Goto City (**C**), and 14, 15, 26, 27 and 30 July at Iki City (**D**–**H**), respectively. Each figure is the result of a backward trajectory analysis of HYSPLIT using the NOAA website: https://www.ready.noaa.gov/HYSPLIT.php.
(TIF)
