# Supplementary figures and images for: Two hidden taxa in the Japanese encephalitis vector mosquito, Culex tritaeniorhynchus, and the potential for long-distance migration from overseas to Japan
Source: PLoS Negl Trop Dis. 2022 Jun 30;16(6):e0010543. doi: 10.1371/journal.pntd.0010543 (PMC9278767; doi:10.1371/journal.pntd.0010543)

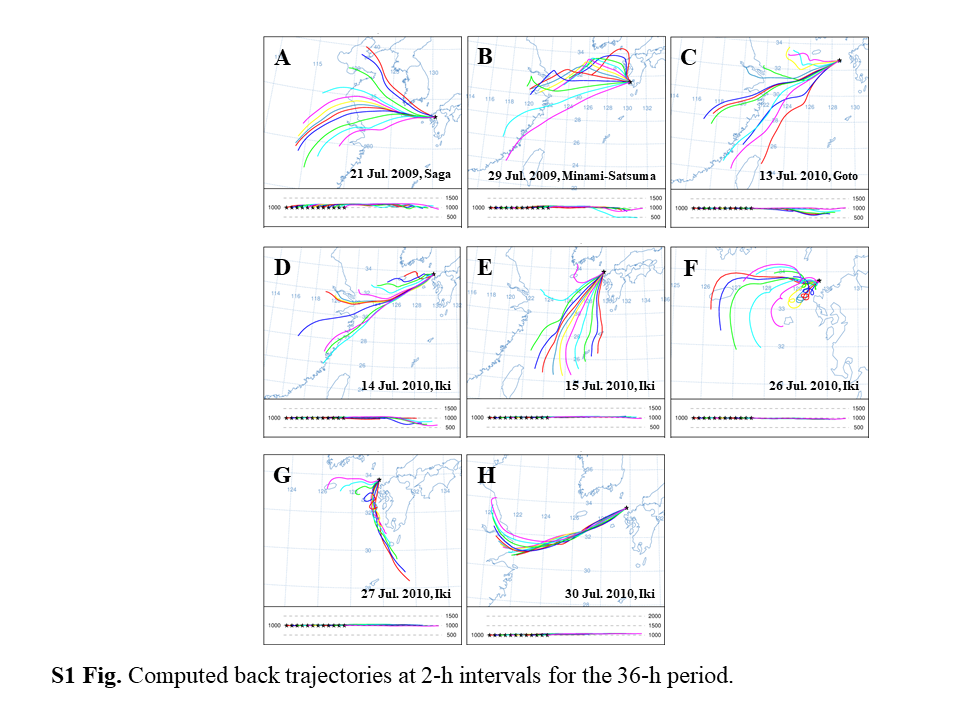

Supplement: S1 Fig — Computing was at 0000 UTC of 21 July 2009 at Saga City (A), 29 July 2009 at Minami-Satsuma City (B), 13 July 2010 at Goto City (C), and 14, 15, 26, 27 and 30 July at Iki City (D–H), respectively. Each figure is the result of a backward trajectory analysis of HYSPLIT using the NOAA website: https://www.ready.noaa.gov/HYSPLIT.php. (TIF) [file pntd.0010543.s002.TIF]
